# Physiological reserve and early recovery across oncologic and non-oncologic abdominal surgery: a prospective multicenter interaction analysis

**DOI:** 10.3389/fonc.2026.1954486

**Published:** 2026-10-15

**Authors:** Sergii Girnyi, Francesco Giovinazzo, Elena Montanari, Eugenia Semeraro, Alessandra Marano, Jaroslaw Skokowski, Tomasz Cwalinski, Mathias Schlögl, Mirko Barone, Archil Aladashvili, Mahmoud Al-Balas, Virginia Boccardi, Mauro Santarelli, Luigi Marano

**Affiliations:** 1 Department of General Surgery and Surgical Oncology, “Saint Wojciech” Hospital, “Nicolaus Copernicus” Health Center, Gdansk, Poland; 2 Department of Surgery, San Camillo Hospital, Treviso, Italy; 3 General and Specialistic Surgery Department, Emergency General Surgery Unit, Azienda Ospedaliero-Universitaria (AOU) Città della Salute e della Scienza di Torino, Turin, Italy; 4 Department of Medicine, Academy of Applied Medical and Social Sciences-Akademia Medycznych I Spolecznych Nauk Stosowanych (AMiSNS), Elblag, Poland; 5 Department for Geriatric Medicine, Clinic Barmelweid AG, Barmelweid, Switzerland; 6 Department of General and Thoracic Surgery, SS. Annunziata University Hospital, Chieti, Italy; 7 Department of Medical, Oral, and Biotechnological Sciences, G. d’Annunzio University of Chieti, Chieti, Italy; 8 Bakhutashvili Institute of Medical Biotechnology, Tbilisi State Medical University, Tbilisi, Georgia; 9 Department of General Surgery, Anesthesia and Urology, Faculty of Medicine, The Hashemite University, Zarqa, Jordan; 10 Section of Gerontology and Geriatrics, Department of Medicine and Surgery, University of Perugia, Perugia, Italy; 11 Department of Surgery, Dnipro State Medical University, Dnipro, Ukraine

**Keywords:** effect modification, frailty, handgrip strength, length of stay, oncologic surgery, physiological reserve

## Abstract

**Background:**

Frailty and low handgrip strength are established markers of limited physiological reserve, but it is uncertain whether their prognostic meaning differs between oncologic and non-oncologic surgery. We examined whether oncologic indication modifies the associations of preoperative frailty and baseline handgrip strength with early postoperative outcomes.

**Methods:**

This secondary analysis used a prospective cohort conducted at participating surgical centers between January 2023 and June 2025. Frailty was classified using the Fried phenotype, and handgrip strength was measured at admission. The primary outcome was prolonged length of stay (LOS >10 days); secondary outcomes were any postoperative complication, major complications, and log-transformed LOS. Effect modification was evaluated using interaction terms in multivariable logistic models adjusted for age and sex. Because no prolonged stays occurred among fit patients in the non-oncologic stratum, categorical frailty models used Firth penalized logistic regression.

**Results:**

Among 222 clinically characterized participants, 118 (53.2%) underwent oncologic surgery. Prolonged LOS occurred in 41/118 (34.7%) oncologic and 7/104 (6.7%) non-oncologic patients. The complete-case primary analysis included 216 patients and 45 events. In an additional ordinal main-effects model, increasing frailty [odds ratio (OR) 2.91 per category, 95% confidence interval (CI) 1.57–5.38], lower handgrip strength (OR 0.95 per kg, 95% CI 0.90–0.99), and oncologic indication (OR 8.97, 95% CI 3.47–23.19) were associated with prolonged LOS. However, categorical frailty-by-oncology interaction was not supported (global *p* = 0.341), nor was handgrip-strength-by-oncology interaction (*p* = 0.739); the joint interaction test was not statistically significant (*p* = 0.525). No convincing effect modification was observed for complications or continuous LOS.

**Conclusions:**

Oncologic surgery was associated with a substantially higher early postoperative burden, while frailty and handgrip strength retained prognostic relevance. No clear evidence of multiplicative interaction by oncologic indication was detected, but clinically meaningful effect modification cannot be excluded. Physiological reserve assessment should therefore be considered across elective abdominal surgery rather than restricted to the oncologic setting.

## 1 Introduction

Frailty is a clinically recognizable state of diminished physiological reserve and impaired homeostatic response to stress. It is conceptually distinct from chronological age, disability, and comorbidity, although these constructs frequently coexist (1, 2). Surgery represents a controlled physiological stressor, and patients with reduced reserve are less able to absorb the metabolic, inflammatory, functional, and organizational demands of the perioperative course. Across general surgical populations, frailty has been associated with complications, longer hospitalization, loss of independence, and mortality (3, 4). The oncologic setting may plausibly alter this relationship. Patients undergoing cancer surgery can enter the perioperative pathway with tumor-related inflammation, nutritional depletion, anemia, cachexia, prior systemic therapy, or radiotherapy exposure; their operations may also involve greater technical complexity and tissue trauma (5–7). These features could increase the clinical consequences of a given degree of baseline vulnerability. Accordingly, oncologic surgery is often included as an adjustment covariate in perioperative outcome models. Adjustment for a main effect, however, does not establish whether the association between physiological reserve and outcome differs across oncologic and non-oncologic contexts. Handgrip strength (HGS) provides a practical, low-cost measure of muscle function and contributes to the weakness domain of the Fried frailty phenotype. Lower preoperative HGS has been associated with longer length of stay (LOS) after abdominal surgery and with morbidity following gastrointestinal cancer surgery (8–10). Nevertheless, evidence is not fully uniform: some colorectal cancer cohorts have not found an independent association between HGS and postoperative complications, and a recent prospective study linked low HGS and frailty to early loss of independence after colorectal cancer resection (11, 12). These findings establish prognostic relevance within oncologic cohorts but do not answer whether the strength of association differs from non-oncologic surgery. Effect modification is a distinct estimand and should be assessed through a formal interaction test rather than by comparing whether associations reach statistical significance in separate subgroups (13, 14). We therefore examined whether oncologic indication modifies the associations of preoperative frailty and baseline HGS with prolonged postoperative hospitalization. We hypothesized that oncologic surgery would amplify the adverse association between reduced physiological reserve and early postoperative outcomes. Any postoperative complication, major complication, and continuous LOS were examined as secondary or exploratory outcomes.

## 2 Materials and methods

### 2.1 Study design and ethical considerations

This was a secondary interaction analysis of a prospective multicenter observational cohort examining perioperative functional measures in adults undergoing elective abdominal surgery. Participants were consecutively enrolled between January 2023 and June 2025 at participating surgical centers. The study was conducted in accordance with the Declaration of Helsinki and Good Clinical Practice. The coordinating ethics committee of AOU Città della Salute e della Scienza di Torino approved the protocol (protocol 0025277, 27 February 2023). All participants provided written informed consent before enrollment. Reporting follows the Strengthening the Reporting of Observational Studies in Epidemiology (STROBE) recommendations (19).

### 2.2 Study population

Adults aged 18 years or older who were scheduled for elective abdominal surgery requiring postoperative hospitalization were eligible. Both oncologic and non-oncologic indications were included. Exclusion criteria were day-case or diagnostic-only procedures, palliative surgery, non-admitted interventions, inability to provide consent, and absence of the baseline measurements required for a given analysis. Of 223 rows in the analytic export, one record contained no usable clinical information and was excluded, leaving 222 clinically characterized participants.

### 2.3 Preoperative physiological reserve

Frailty was assessed at hospital admission using an assessment based on the five domains of the Fried phenotype (2). Unintentional weight loss was defined as self-reported involuntary loss of >4.5 kg during the preceding year. Exhaustion was defined as fatigue reported on at least 3 days per week. Reduced physical activity was assessed clinically on the basis of self-reported limitations in habitual mobility or reduced participation in usual physical activities. Slowness was assessed clinically from reduced habitual walking performance, mobility limitation, or visibly impaired gait. For the weakness domain, the binary item recorded during the parent cohort’s baseline assessment was retained; the procedure used to measure baseline HGS is described below. The recorded frailty classification was retained for this secondary analysis, using the categories fit (0 criteria), pre-frail (1–2 criteria), and frail (3–5 criteria). Five domains were evaluated: unintentional weight loss, self-reported exhaustion, low physical activity, slowness, and weakness. Participants were classified as fit (0 criteria), pre-frail (1–2 criteria), or frail (3–5 criteria). Frailty was treated as a three-level categorical predictor in the primary interaction analyses, using fit as the reference category and separate indicators for pre-frail and frail status. This specification did not impose equal effects across adjacent categories. Ordinal coding (fit = 0, pre-frail = 1, and frail = 2) was used to summarize the overall trend and in sensitivity analyses. In logistic models, this coding assumes an equal change in log odds per category increment; it does not establish equal clinical distances between categories.

Baseline HGS was measured before surgery at hospital admission with a calibrated digital dynamometer (EH101, Deyard, Shenzhen, China), following American Society of Hand Therapists recommendations (15). Participants were seated with the shoulder adducted, the elbow flexed at 90°, and the wrist in slight extension. Following three familiarization contractions, participants performed three maximal voluntary isometric contractions with the dominant hand, and the highest value was retained for analysis. HGS was analyzed continuously in kilograms. For interaction models, HGS was mean-centered at 25.11 kg to make the oncologic main-effect coefficient interpretable at the cohort mean.

### 2.4 Surgical context and outcomes

The candidate effect modifier was surgical indication, categorized prospectively as oncologic or non-oncologic. The primary outcome was prolonged postoperative LOS (>10 days). This cutoff was selected after examining the parent cohort’s LOS distribution, approximately corresponding to its 75th percentile, and was subsequently used in reports from that cohort (25, 28). We retained this definition for consistency with those analyses. Its clinical interpretation is an extended inpatient course and associated hospitalization burden within this mixed surgical population, reflecting recovery, complications, discharge readiness, social support, and institutional processes. Postoperative complications were graded according to the Clavien–Dindo classification (16). Secondary outcomes were any postoperative complication and continuous LOS, analyzed after natural-log transformation as log(LOS + 1). Major complications (Clavien–Dindo grade III or higher) were analyzed exploratorily because of the small number of events.

### 2.5 Statistical analysis

Continuous variables are summarized as mean with standard deviation (SD) or median with interquartile range (IQR), and categorical variables are summarized as counts and percentages. Oncologic and non-oncologic groups were compared using Welch’s *t*-test for approximately symmetric continuous variables, the Mann–Whitney *U* test for LOS, and Pearson chi-square or Fisher exact tests for categorical variables, as appropriate. These comparisons were descriptive and were not used to select model covariates. The primary query was multiplicative effect modification on the odds ratio (OR) scale. Three adjusted models were specified: (1) frailty category, oncologic indication, and frailty-by-oncology terms; (2) centered HGS, oncologic indication, and HGS-by-oncology; and (3) a joint model containing both interactions. All primary interaction models included age, sex, frailty, HGS, and oncologic indication so that the two reserve measures were mutually adjusted. Frailty was entered as two indicator variables with fit as the reference category. The global frailty interaction was evaluated jointly at two degrees of freedom. No prolonged stays occurred among fit patients in the non-oncologic stratum, producing complete separation in conventional categorical logistic regression. Primary categorical models therefore used Firth penalized logistic regression, which provides finite bias-reduced estimates under separation (17). Wald 95% confidence intervals (CIs) and global Wald tests are reported. Adjusted probabilities were obtained from the joint penalized model by standardization over the complete-case covariate distribution. Sensitivity analyses treated frailty as an ordinal exposure and fitted conventional maximum-likelihood logistic models, with likelihood-ratio tests for the interaction terms. Age- and sex-adjusted stratified models were also fitted separately in oncologic and non-oncologic participants; these estimates were presented for clinical interpretation but were not substituted for formal interaction tests. Firth ordinal regression was used as a further sparse-data sensitivity analysis. The same interaction framework was applied to any complication and major complications. Log-transformed LOS was analyzed using multivariable linear regression. Analyses used complete cases; no imputation was performed because baseline HGS was missing for 6/222 (2.7%) participants. The primary complete-case sample comprised 216 patients. The cohort size was fixed by the parent study. The binary prolonged-LOS indicator was available for all 222 clinically characterized participants, whereas exact LOS in days was available for 221; the one participant with a recorded binary indicator but missing exact LOS contributed to binary-outcome analyses and was excluded from continuous-LOS analyses. No interaction-specific sample-size calculation was prespecified. Interaction analyses were therefore interpreted using effect estimates, CIs, event counts, and consistency across analyses rather than *p*-values alone; small-sample interaction tests can be unstable (18). Spearman correlations between frailty category and HGS were calculated overall and by oncologic indication, and multicollinearity was assessed using variance inflation factors (VIFs). For prolonged LOS, we compared Firth logistic models containing frailty alone, HGS alone, or both measures, using the same 216 complete cases. Every model included age, sex, and oncologic indication, with frailty modeled categorically. Interaction analyses were also repeated without mutual adjustment for the other reserve measure. All tests were two-sided with alpha = 0.05. Analyses were performed using IBM SPSS Statistics, version 32.0 (IBM Corp., Armonk, NY, USA), for descriptive statistics, between-group comparisons, and conventional logistic regression models. Firth penalized logistic regression, two-degree-of-freedom joint Wald tests of interaction terms, and covariate-standardized predicted probabilities were implemented in R, version 4.6.1 (R Foundation for Statistical Computing, Vienna, Austria), using the logistf package, version 1.26.1.

## 3 Results

### 3.1 Cohort characteristics by oncologic indication

The analytic cohort included 222 clinically characterized participants: 118 (53.2%) underwent oncologic surgery and 104 (46.8%) underwent non-oncologic surgery. Patients in the oncologic group were older (67.2 ± 12.9 vs. 61.8 ± 17.0 years; *p* = 0.010) and more often pre-frail or frail (*p* = 0.039), while baseline HGS was similar (24.86 ± 11.03 vs. 25.39 ± 11.43 kg; *p* = 0.729). Sex distribution did not differ materially between groups (50.0% vs. 58.7% male; *p* = 0.197) (**Table 1**). Early postoperative burden was substantially greater in the oncologic group. Median LOS was 8 days (IQR 6–12) compared with 3 days (IQR 2–5; *p* < 0.001). Prolonged LOS occurred in 41/118 (34.7%) oncologic patients and 7/104 (6.7%) non-oncologic patients (*p* < 0.001). Any complication occurred in 49.2% and 21.2%, respectively (*p* < 0.001), whereas major complications were uncommon and did not differ statistically (9.3% vs. 3.8%; *p* = 0.117).

**Table 1:** Characteristics and outcomes according to surgical indication.

Variable	Non-oncologic	Oncologic	*p*-value
Participants, *n*	104	118	–
Age, years, mean ± SD	61.8 ± 17.0	67.2 ± 12.9	0.010
Male sex, *n* (%)	61 (58.7)	59 (50.0)	0.197
Frailty status, *n* (%)			0.039
Fit	48 (46.2)	35 (29.7)	
Pre-frail	37 (35.6)	53 (44.9)	
Frail	19 (18.3)	30 (25.4)	
Baseline HGS, kg, mean ± SD	25.39 ± 11.43	24.86 ± 11.03	0.729
LOS, days, median (IQR)	3 (2-5)	8 (6-12)	<0.001
LOS >10 days, *n* (%)	7 (6.7)	41 (34.7)	<0.001
Any complication, *n* (%)	22 (21.2)	58 (49.2)	<0.001
Major complication, *n* (%)	4 (3.8)	11 (9.3)	0.117

HGS, handgrip strength; IQR, interquartile range; LOS, length of stay; SD, standard deviation. Major complications were Clavien–Dindo grade III or higher. HGS was available for 104 non-oncologic and 112 oncologic participants; raw LOS was available for 104 and 117, respectively. *p*-values are descriptive.

### 3.2 Main associations with prolonged length of stay

The primary complete-case analysis included 216 patients and 45 prolonged-LOS events: 38/112 in the oncologic stratum and 7/104 in the non-oncologic stratum. In the additional adjusted ordinal main-effects model, each increment from fit to pre-frail or from pre-frail to frail was associated with higher odds of prolonged LOS (OR 2.91, 95% CI 1.57–5.38; *p* < 0.001). Higher HGS was associated with lower odds (OR 0.947 per kg, 95% CI 0.903–0.993; *p* = 0.025). Oncologic indication was associated with prolonged LOS after adjustment for age, sex, frailty, and HGS (OR 8.97, 95% CI 3.47–23.19; *p* < 0.001). Male sex was also associated with higher odds (OR 5.54, 95% CI 1.97–15.59; *p* = 0.001), whereas the association with age was not statistically significant (**Table 2**).

**Table 2:** Adjusted associations and interaction tests for prolonged length of stay.

Model/term	Adjusted OR	95% CI	*p*-value	Global interaction *p*
Additional ordinal main-effects model				
Age, per 10 years	0.775	0.560–1.073	0.124	
Male sex	5.544	1.972–15.587	0.001	
Frailty, per category	2.910	1.575–5.377	<0.001	
Baseline HGS, per kg	0.947	0.903–0.993	0.025	
Oncologic indication	8.965	3.466–23.187	<0.001	
Categorical frailty interaction model (Firth)				0.341
Pre-frail × oncologic	0.099	0.004–2.301	0.150	
Frail × oncologic	0.112	0.005–2.752	0.180	
HGS interaction model (Firth)				0.739
HGS × oncologic, per kg	1.014	0.936–1.097	0.739	
Joint interaction model (Firth)				0.525
Pre-frail × oncologic	0.100	0.005–2.135	0.140	
Frail × oncologic	0.116	0.004–3.265	0.206	
HGS × oncologic, per kg	1.000	0.914–1.094	0.994	

CI, confidence interval; Firth, bias-reduced penalized logistic regression; HGS, handgrip strength; OR, odds ratio. All models included age, sex, frailty, HGS, and oncologic indication. HGS was centered at 25.11 kg. Fit status and non-oncologic surgery were reference categories. Global *p*-values jointly test all interaction terms in the indicated model.

### 3.3 Effect modification by oncologic indication

In the Firth model containing categorical frailty interactions, the interaction ORs were 0.10 (95% CI 0.004–2.30) for pre-frail status and 0.11 (95% CI 0.005–2.75) for frail status relative to fit; the two-degree-of-freedom global interaction test was not significant (*p* = 0.341). The HGS-by-oncology interaction OR was 1.014 per kg (95% CI 0.936–1.097; *p* = 0.739). In the joint model, none of the interaction terms was supported and the three-degree-of-freedom global test yielded *p* = 0.525 (**Table 2**). Standardized probabilities from the joint penalized model illustrate the large main effect of surgical context and increasing risk across frailty categories, together with substantial uncertainty in the non-oncologic stratum (**Figure 1**). At mean HGS, adjusted prolonged-LOS probabilities for fit, pre-frail, and frail participants were 1.0%, 11.9%, and 26.9% in non-oncologic surgery and 26.6%, 33.8%, and 59.6% in oncologic surgery. Across the observed HGS range, the probability curves declined in both groups without evidence of divergent HGS slopes on the log-odds scale (**Figure 2**).

**Figure 1:**
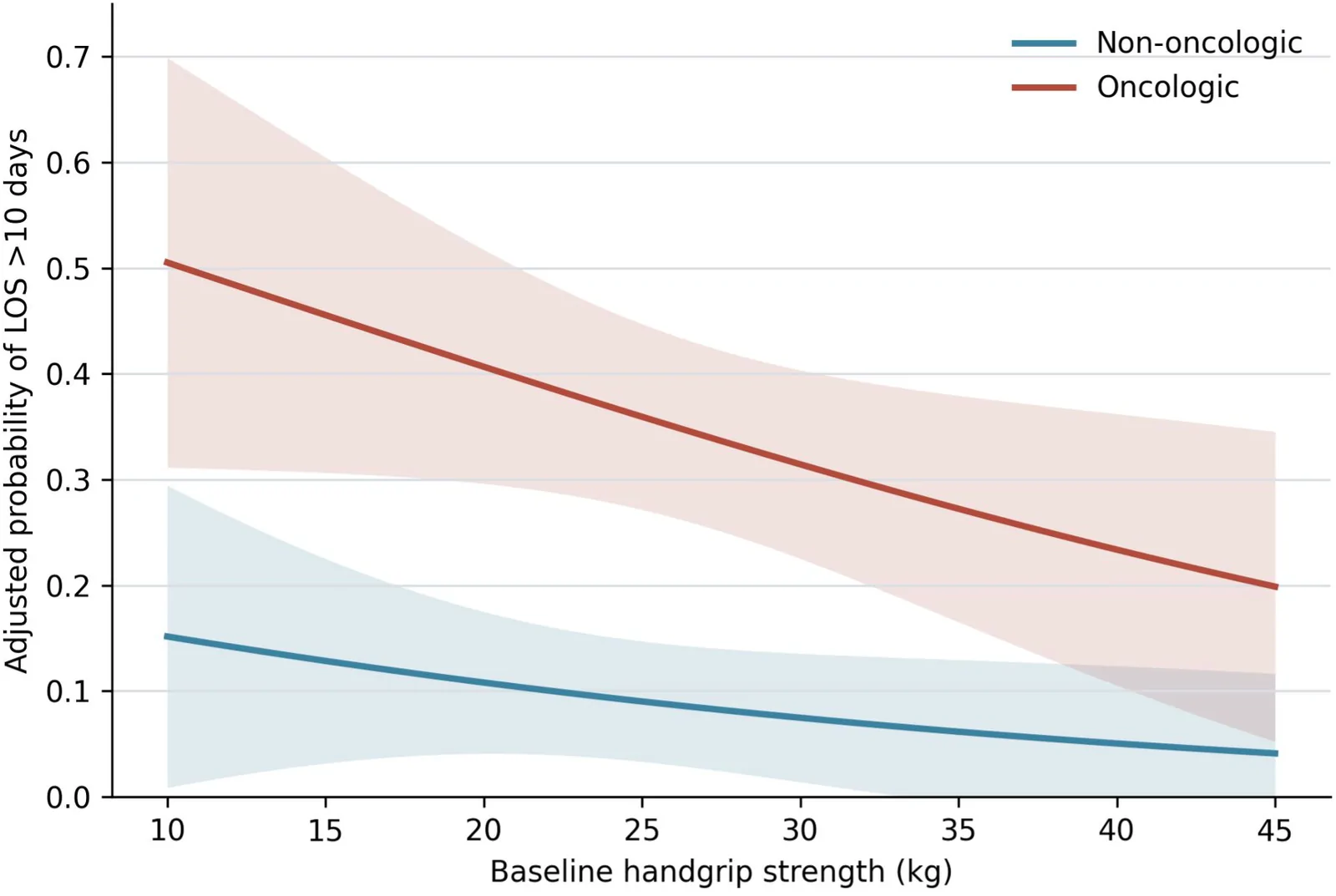
Adjusted probability of prolonged length of stay across frailty categories by surgical indication. Probabilities and 95% confidence intervals were standardized from the joint Firth penalized logistic model over the complete-case age and sex distribution, with handgrip strength fixed at the cohort mean (25.11 kg).

**Figure 2:**
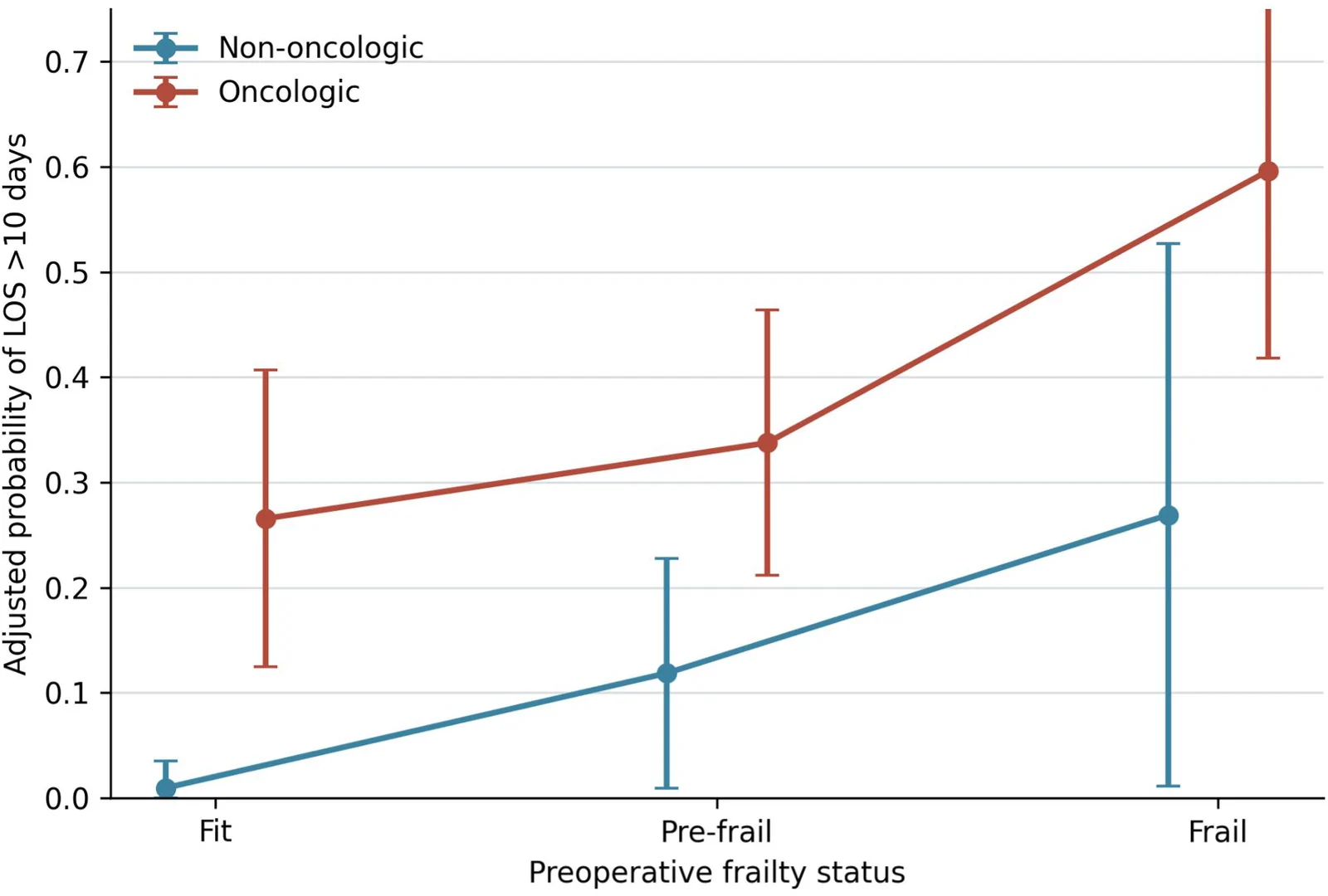
Adjusted probability of prolonged length of stay across baseline handgrip strength by surgical indication. Curves and 95% confidence bands were standardized from the joint Firth penalized logistic model over the complete-case age, sex, and frailty distribution.

### 3.4 Stratified and sensitivity analyses

In ordinal stratified models, increasing frailty was associated with prolonged LOS in both non-oncologic (OR 8.27 per category, 95% CI 1.34–50.97; *p* = 0.023) and oncologic surgery (OR 2.43, 95% CI 1.22–4.85; *p* = 0.011). HGS was not associated with the outcome in the non-oncologic stratum (OR 0.995 per kg, 95% CI 0.894–1.107; *p* = 0.926) but was inversely associated in the oncologic stratum (OR 0.928, 95% CI 0.874–0.985; *p* = 0.013) (**Figure 3**). These differences in stratum-specific statistical significance did not constitute evidence of interaction. Penalized categorical stratified estimates were imprecise, particularly in the non-oncologic group, because all seven events occurred among pre-frail or frail patients (**Table 3**). Treating frailty as ordinal did not change the inference: frailty-by-oncology interaction *p* = 0.124, HGS-by-oncology interaction *p* = 0.691, and joint interaction *p* = 0.282. The Firth ordinal sensitivity analysis estimated an interaction OR of 0.41 for frailty-by-oncology (95% CI 0.12–1.48; *p* = 0.175). Frailty and HGS were inversely correlated (Spearman ρ = −0.330 overall, −0.401 in non-oncologic surgery, and −0.266 in oncologic surgery). VIFs ranged from 1.07 to 2.09. Mutual adjustment attenuated the associations of frailty and HGS with prolonged LOS without changing their direction (**Table 4**). Interaction tests remained nonsignificant without mutual adjustment (frailty-by-oncology *p* = 0.320; HGS-by-oncology *p* = 0.514), although CIs remained wide.

**Figure 3:**
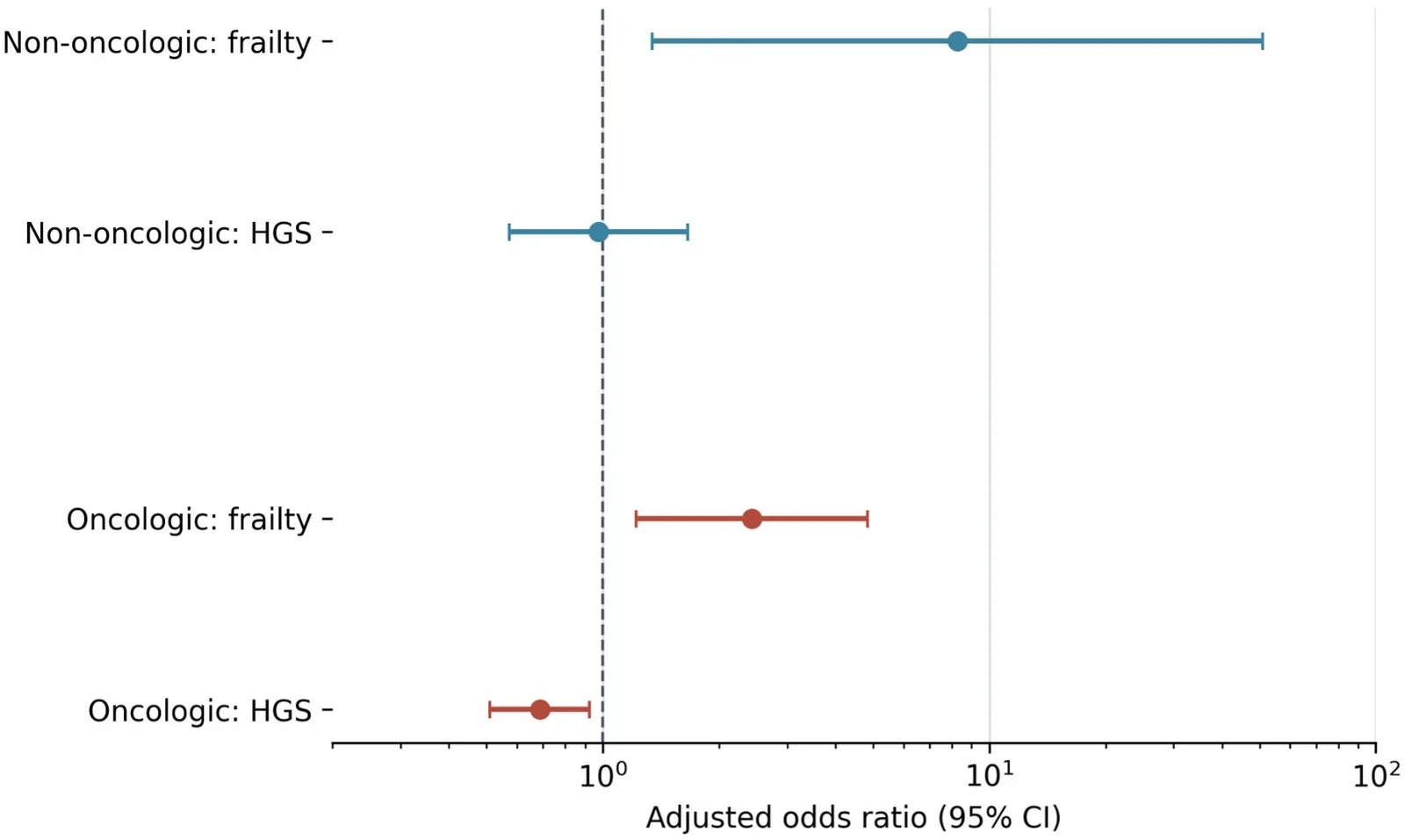
Stratified associations of frailty and handgrip strength with prolonged length of stay in sensitivity analyses. Odds ratios are from binary logistic regression models including age; sex; frailty coded as 0, 1, or 2; and handgrip strength. Frailty is expressed per one-category increase; handgrip strength is expressed per 5-kg increase. Stratum-specific statistical significance should not be interpreted as evidence of interaction.

**Table 3:** Stratified Firth logistic models for prolonged length of stay.

Stratum/term	Adjusted OR	95% CI	*p*-value	Frailty global P
Non-oncologic (*n* = 104; 7 events)				0.083
Age, per 10 years	0.782	0.440–1.393	0.404	
Male sex	1.266	0.206–7.758	0.799	
Pre-frail vs. fit	11.250	0.697–181.659	0.088	
Frail vs. fit	39.727	1.551–1017.360	0.026	
HGS, per kg	0.984	0.896–1.082	0.745	
Oncologic (*n* = 112; 38 events)				0.038
Age, per 10 years	0.775	0.527–1.140	0.195	
Male sex	8.668	2.409–31.192	<0.001	
Pre-frail vs. fit	1.543	0.528–4.505	0.428	
Frail vs. fit	5.380	1.407–20.572	0.014	
HGS, per kg	0.938	0.884–0.995	0.033	

CI, confidence interval; HGS, handgrip strength; OR, odds ratio. Models were adjusted for age, sex, frailty category, and HGS. Global *p*-values jointly test pre-frail and frail categories. The very wide non-oncologic intervals reflect seven events and no events among fit participants.

**Table 4:** Frailty and handgrip strength correlations and model comparisons for prolonged length of stay.

A. Correlation between frailty category and HGS			
Population	Spearman ρ	95% CI	*p*-value
Overall (*n* = 216)	-0.330	−0.450 to −0.202	<0.001
Non-oncologic (*n* = 104)	−0.401	−0.558 to −0.222	<0.001
Oncologic (*n* = 112)	−0.266	−0.441 to −0.075	0.005
B. Firth logistic models for prolonged LOS			
Predictor	Frailty only	HGS only	Frailty and HGS
Pre-frail versus fit	1.95 (0.76–4.99) *p* = 0.166	—	2.21 (0.84–5.81) *p* = 0.107
Frail versus fit	8.43 (2.61–27.20) *p* < 0.001	—	7.59 (2.32–24.82) *p* < 0.001
HGS per 1 kg	—	0.943 (0.900–0.987) *p* = 0.012	0.952 (0.908–0.999) *p* = 0.045
Oncologic versus non-oncologic	6.92 (2.89–16.61) *p* < 0.001	8.24 (3.40–19.97) *p* < 0.001	8.02 (3.23–19.93) *p* < 0.001

CI, confidence interval; HGS, handgrip strength; LOS, length of stay; OR, odds ratio; VIF, variance inflation factor.

Panel A: Frailty was ordered as fit, pre-frail, and frail. Spearman correlations do not assume equal clinical distances between categories. CIs are percentile intervals from 10,000 participant-level bootstrap resamples within each population; two-sided *p*-values use 99,999 random permutations with a plus-one correction.

Panel B: Values are adjusted ORs (95% Wald CIs) and *p*-values. Every model includes age, sex, and oncologic indication and uses the same 216 participants and 45 prolonged-LOS events (>10 days). “Only” refers to the reserve measure included, not to univariable analysis. Frailty is categorical with fit as the reference; HGS is centered at 25.11 kg. Models contain main effects without interaction terms. An em dash denotes a predictor omitted from that model. Global two-degree-of-freedom Wald *p*-values for frailty are 0.001 in the frailty-only model and 0.003 in the combined model.

VIFs in the combined main-effects design were 1.57 for age, 1.79 for sex, 1.07 for oncologic indication, 1.36 for pre-frail status, 1.75 for frail status, and 2.09 for HGS. The intercept was excluded from these diagnostics.

### 3.5 Secondary outcomes

Among all 222 clinically characterized participants, any postoperative complication occurred in 80 and major complications occurred in 15. Complete-case interaction analyses included 216 participants, with 77 any-complication events and 14 major-complication events. For any complication, there was no clear frailty-by-oncology interaction (*p* = 0.134), HGS-by-oncology interaction (*p* = 0.230), or joint interaction (*p* = 0.264). Major-complication estimates were highly imprecise and showed no interaction signal (*p* = 0.679, *p* = 0.816, and joint *p* = 0.915, respectively). In the continuous LOS analysis (*n* = 215), neither interaction was supported (frailty *p* = 0.843; HGS *p* = 0.876; joint *p* = 0.976).

## 4 Discussion

In this prospective multicenter cohort, preoperative physiological reserve and oncologic surgical context were each associated with early postoperative hospitalization burden, but the central hypothesis of effect modification was not supported. Frailty, lower baseline HGS, and oncologic indication were associated with prolonged LOS in the main-effects model. Formal interaction testing, however, did not demonstrate that oncologic indication amplified or otherwise altered the associations of frailty or HGS with prolonged LOS. The same conclusion was obtained with categorical penalized models, ordinal sensitivity analyses, and secondary outcomes. This distinction between a strong main effect and effect modification is clinically important. Oncologic surgery identified a group with markedly higher absolute event rates, longer hospitalization, and more complications. Cancer-associated inflammation, nutritional depletion, acute muscle catabolism, and procedural complexity provide plausible mechanisms for this increased baseline burden (5–7). Nevertheless, a higher outcome rate in the oncologic group does not imply that the relative prognostic meaning of a given frailty grade or HGS value must differ. In our data, oncologic indication was associated with a higher risk level without producing a detectable multiplicative interaction with baseline reserve. The main associations are consistent with a substantial literature showing that frailty predicts complications, prolonged hospitalization, functional decline, and mortality after general and cancer surgery (3–5, 20–28). Preoperative HGS has likewise been associated with postoperative morbidity in gastric and gastrointestinal cancer cohorts (9, 10), while null findings have also been reported in colorectal cancer surgery (11). The recent study by Zhao and colleagues linked both low HGS and frailty to early loss of independence after colorectal cancer resection (12). Our results extend this evidence by asking a different question: not whether reserve predicts outcome within cancer surgery, but whether the reserve–outcome association differs from that observed in non-oncologic abdominal surgery. The stratified estimates require careful interpretation. HGS was statistically associated with prolonged LOS only in the oncologic stratum, whereas the frailty OR appeared numerically larger in non-oncologic surgery. Neither pattern establishes heterogeneity, because the correct comparison is the interaction term rather than the significance level within each subgroup (14). The non-oncologic stratum contained only seven prolonged-stay events and no events among fit patients, leading to wide CIs and complete separation. Penalization yielded finite estimates but cannot create information absent from sparse cells. Thus, the data argue against claiming oncologic amplification, but they also do not establish equivalence of effects across contexts. On the absolute-risk scale, adjusted probabilities increased with frailty in both groups. The oncologic group had higher predicted risk at every frailty level, while uncertainty was greatest in the non-oncologic fit and frail categories. Effect modification is scale-dependent (13); therefore, the lack of evidence of multiplicative interaction should not be interpreted as absence of all clinically relevant heterogeneity. Rather, the clinically dominant pattern was a higher baseline risk associated with oncologic surgery superimposed on a broadly adverse gradient of reduced reserve. These findings have practical implications. Preoperative frailty and HGS assessment should not be reserved for patients with cancer. Reduced reserve was clinically relevant across elective abdominal surgery, although the precision of subgroup estimates differed. Oncologic indication should be considered alongside, not as a replacement for, direct measurement of physiological reserve. Patients undergoing cancer surgery may warrant intensified nutritional, functional, and discharge planning because of their higher absolute risk, while fit, pre-frail, and frail status can still inform shared decision-making and perioperative optimization in both contexts. Strengths of this study include prospective data collection, direct preoperative assessment of both a multidomain frailty phenotype and objective muscle strength, inclusion of oncologic and non-oncologic procedures within the same cohort, and explicit testing of interaction rather than inference from subgroup-specific *p*-values. The analysis also addressed complete separation with Firth penalization and examined consistency across binary and continuous outcomes. Several limitations temper interpretation. First, this was a secondary, non-prespecified interaction analysis, and no interaction-specific sample-size calculation was performed. With only seven prolonged-LOS events in the non-oncologic group, interaction estimates were imprecise, with wide CIs. The findings should therefore be regarded as inconclusive regarding effect modification and do not exclude clinically meaningful interactions. Second, oncologic indication is a broad proxy for surgical context rather than a direct measure of stress intensity. Tumor site and stage, surgical site and procedure, chemotherapy status, neoadjuvant treatment, operative approach, duration, blood loss, multivisceral resection, nutritional status, comorbidity, and postoperative care pathways were not included in the adjusted models; residual confounding and clinical heterogeneity are therefore likely. Accordingly, the estimates should be interpreted as associations adjusted for the measured covariates and should not be interpreted as isolating the effect of the tumor itself. Third, center identifiers were not included in the analytic dataset, and the analyses did not account for center effects or within-center correlation. The reported associations may therefore partly reflect differences in center-specific discharge criteria and perioperative care pathways. CIs and *p*-values also do not account for within-center clustering. Fourth, LOS is shaped by clinical recovery and institutional or social determinants. The >10-day cutoff is cohort dependent, and its clinical meaning may vary with the participating centers, procedural case mix, discharge practices, socioeconomic circumstances, and availability of post-discharge support. It should therefore be interpreted as an operational measure of prolonged hospitalization within this cohort rather than a universal threshold of delayed recovery.

Fifth, baseline HGS was missing in six participants, and complete-case analysis assumes that this limited missingness did not introduce material selection bias. Finally, the findings apply to elective admitted abdominal surgery and should not be extrapolated to emergency, palliative, or ambulatory procedures. Future studies should prespecify interaction hypotheses, recruit sufficient events within each surgical context, retain center identifiers, and quantify operative stress more granularly. Repeated functional measures, inflammatory and nutritional biomarkers, and patient-centered endpoints such as loss of independence and quality of life may clarify whether particular oncologic exposures, not oncologic indication in aggregate, modify the consequences of reduced physiological reserve.

## 5 Conclusion

Oncologic indication was associated with a substantially greater early postoperative burden, while frailty and lower baseline HGS were associated with prolonged hospitalization. No clear evidence of multiplicative interaction by oncologic indication was detected, but clinically meaningful effect modification cannot be excluded. The findings support assessment of physiological reserve across elective abdominal surgery and argue against inferring oncologic-specific amplification from subgroup associations alone. Because interaction estimates were imprecise, larger prespecified studies with granular measures of oncologic and operative stress are required.

## Data Availability

The datasets presented in this article are not readily available because confidentiality and data protection. Requests to access the datasets should be directed to not applicable.
